# Supplementary figures and images for: On limitations of uniplex networks for modeling multiplex contagion
Source: PLoS One. 2023 Jan 20;18(1):e0279345. doi: 10.1371/journal.pone.0279345 (PMC9858459; doi:10.1371/journal.pone.0279345)

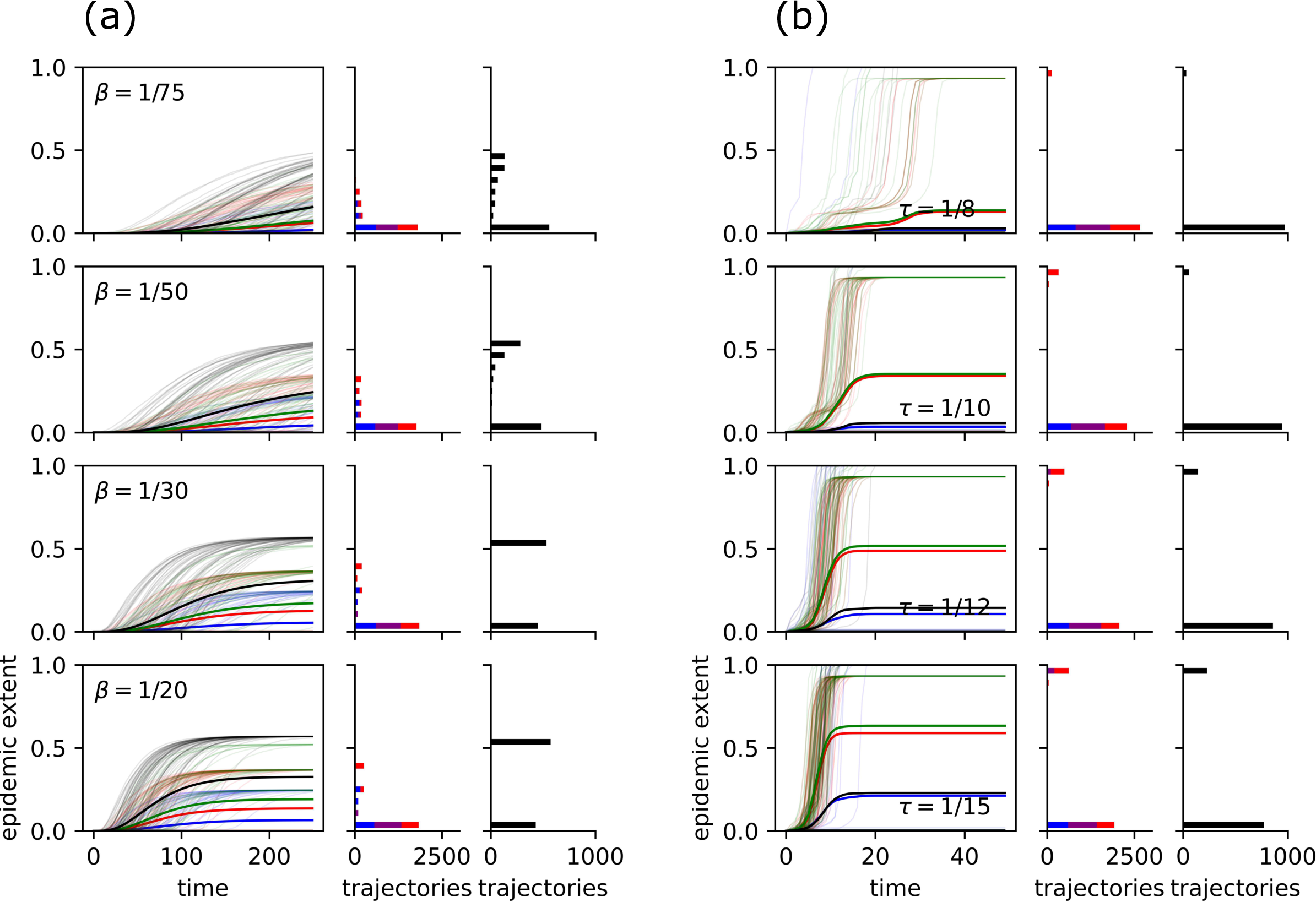

Supplement: S1 Fig — We show that our results hold for the full (a) Project 90 and (b) JOAPP data sets, not just the largest component of the multiplexed data. In contrast to Figs 2b and 3b in the main text, the multiplexed data set is no longer fully connected, leading to some epidemic trajectories that reach very few nodes, resulting in the bimodal distribution of epidemic extents. For the Project 90 data set, we see in S1(a) Fig that the relative epidemic extents are preserved when compared with the epidemic extents of the largest connected component of the multiplexed data. This is not the case with the JOAPP data set in S1(b) Fig, but as discussed in the main text, this is to be expected due to the two competing factors, network density and connectedness, that determine the epidemic extent for the threshold contagion process. For more details on these plots, see Figs 2b and 3b in the main text. (TIF) [file pone.0279345.s001.tif]

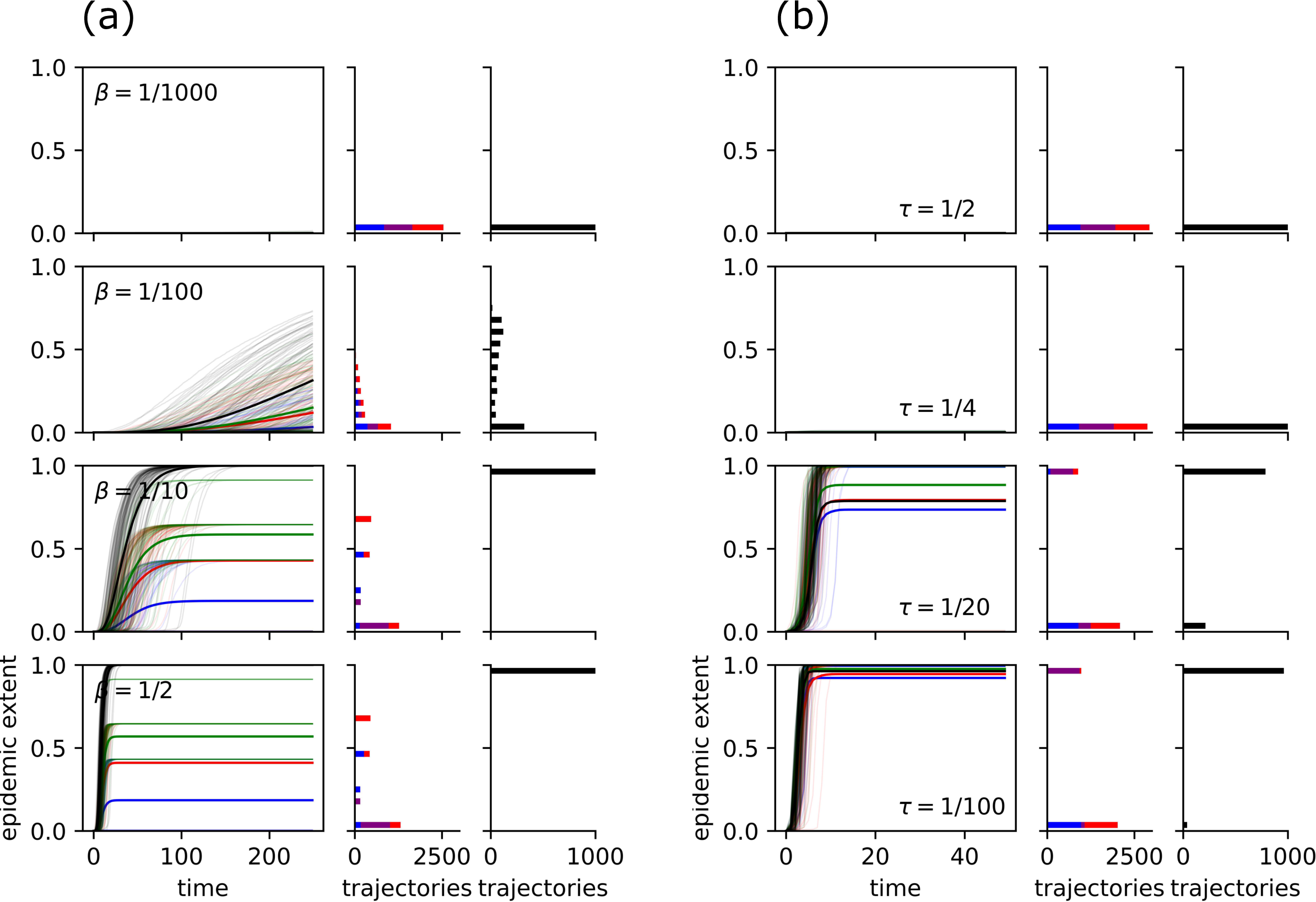

Supplement: S2 Fig — We simulate each contagion process for a wider range of parameters for (a) the Project 90 data set and (b) the JOAPP data set to verify that our results are not dependent on the choice of parameters. For the SI model on the Project 90 data set in S2(a) Fig, we notice the same differences in epidemic extents, albeit on different time scales. This should be anticipated; if we plot the epidemic extent with respect to βt, the average of each time series should be the same. For the threshold process on the JOAPP data set in S2(b) Fig, there are parameter values where the epidemic extents are trivially the same. First, if we choose a threshold greater than the maximum possible fraction of infected neighbors that the network structure and number of seed nodes allow, then contagion will never occur. Second, if the threshold is low enough and the multiplex and uniplex representations are all fully connected, then the entire population will be infected no matter the data representation. For more details on these plots, see Figs 2b and 3b in the main text. (TIF) [file pone.0279345.s002.tif]

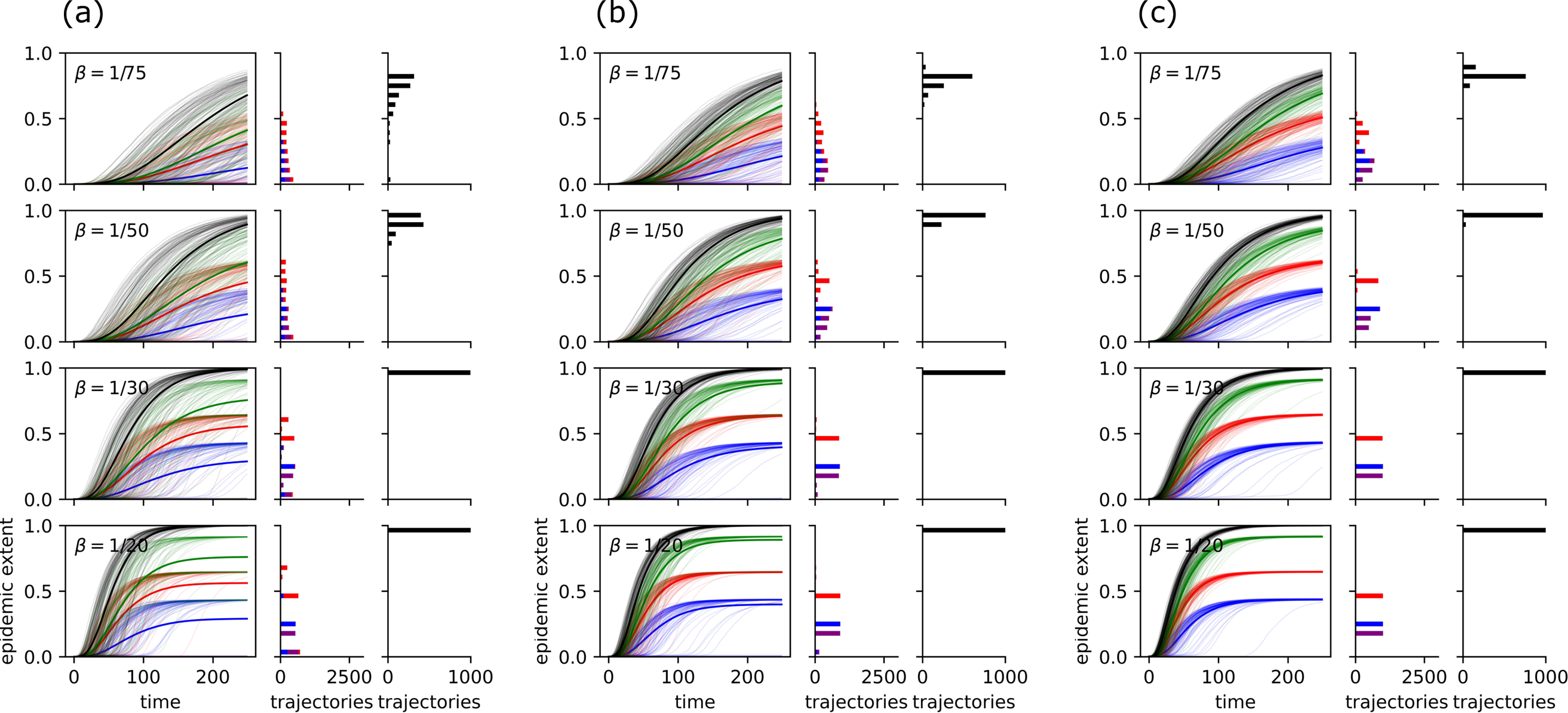

Supplement: S3 Fig — We show that our results hold for (a) 2, (b) 5, and (c) 10 seeds as well as a single seed node as presented in the main text. In S3 Fig, we see that the relative differences in the epidemic extents are preserved. The largest difference is that with a larger number of seed nodes, the likelihood that there is an epidemic trajectory that spreads to very few nodes is much smaller as can be seen in the figure. For more details on these plots, see Figs 2b and 3b in the main text. (TIF) [file pone.0279345.s003.tif]

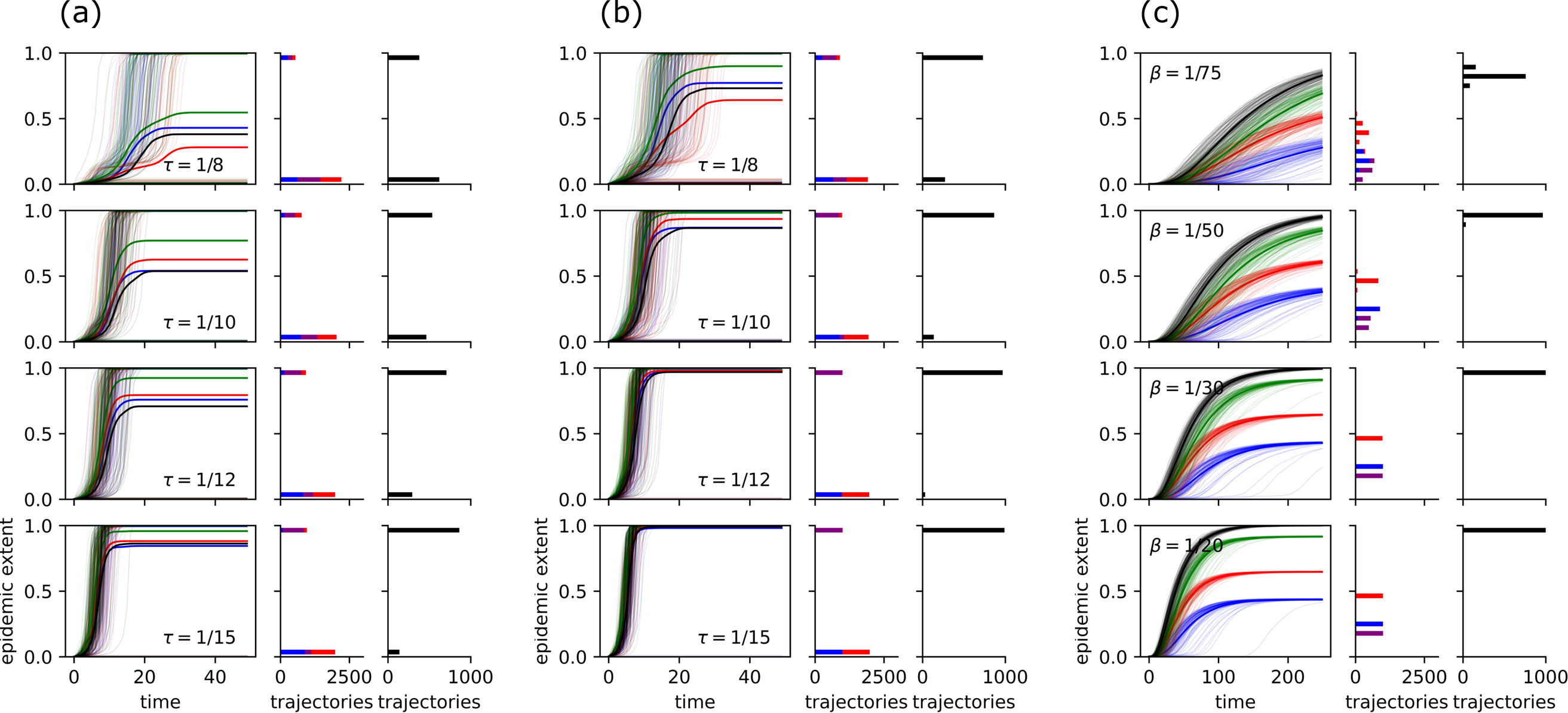

Supplement: S4 Fig — We show that our results hold for (a) 2, (b) 5, and (c) 10 seeds as well as a single seed node as presented in the main text. In S4 Fig, we see that, as in S3 Fig, there is a smaller chance of trajectories dying out. In addition, we see that increasing the number of seed nodes effectively raises the maximum threshold for which the contagion will spread to the entire network (for example, τ = 1/10 in S4 Fig) and lead to trivial results as discussed prior. For more details on these plots, see Figs 2b and 3b in the main text. (TIF) [file pone.0279345.s004.tif]

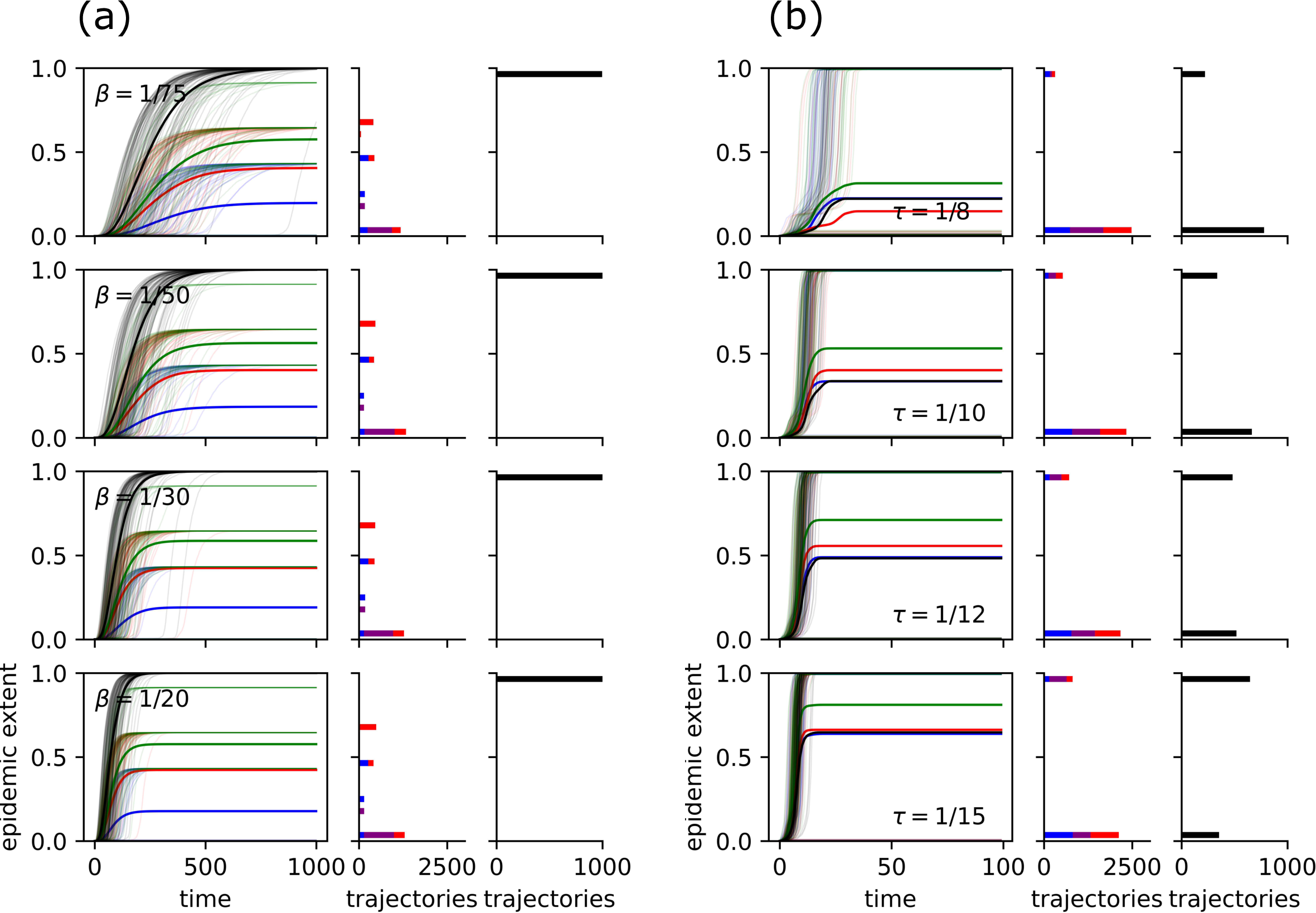

Supplement: S5 Fig — We run the simulations for a long enough time to remove any temporal censoring for (a) smaller values of β for the SI model on the Project 90 data set and for (b) larger values of τ for the threshold model on the JOAPP data set. In S5 Fig, we see that the epidemic extents are consistent with our results in the main text. For the SI model, this should be expected as explained prior; rescaling time by the infection probability should yield very similar epidemic responses in expectation. For more details on these plots, see Figs 2b and 3b in the main text. (TIF) [file pone.0279345.s005.tif]
